# Impact of the 12-gene recurrence score assay on deciding adjuvant chemotherapy for stage II and IIIA/B colon cancer: the SUNRISE-DI study

**DOI:** 10.1016/j.esmoop.2021.100146

**Published:** 2021-05-10

**Authors:** E. Oki, J. Watanabe, T. Sato, Y. Kagawa, Y. Kuboki, M. Ikeda, H. Ueno, T. Kato, T. Kusumoto, T. Masuishi, K. Yamaguchi, A. Kanazawa, T. Nishina, H. Uetake, T. Yamanaka, T. Yoshino

**Affiliations:** 1 Department of Surgery and Science, Kyushu University Graduate School of Medical Sciences, Fukuoka, Japan; 2 Department of Surgery, Gastroenterological Center, Yokohama City University Medical Center, Yokohama, Japan; 3 Department of Colorectal Surgery, Kitasato University Hospital, Kanagawa, Japan; 4 Department of Surgery, Kansai Rosa Hospital, Hyogo, Japan; 5 Department of Gastroenterology and Gastrointestinal Oncology, National Cancer Center Hospital East, Chiba, Japan; 6 Department of Surgery, Hyogo College of Medicine, Hyogo, Japan; 7 Department of Surgery, National Defense Medical College, Saitama, Japan; 8 Department of Surgery, NHO Osaka National Hospital, Osaka, Japan; 9 Department of Gastroenterological Surgery, NHO National Kyushu Medical Center, Fukuoka, Japan; 10 Department of Clinical Oncology, Aichi Cancer Center Hospital, Aichi, Japan; 11 Department of Gastroenterological Chemotherapy, Cancer Institute Hospital of the Japanese Foundation for Cancer Research, Tokyo, Japan; 12 Department of Gastroenterological Surgery, Shimane Prefectural Central Hospital, Shimane, Japan; 13 Department of Gastrointestinal Medical Oncology, NHO Shikoku Cancer Center, Ehime, Japan; 14 Department of Specialized Surgeries, Tokyo Medical and Dental University, Tokyo, Japan; 15 Department of Biostatistics, Yokohama City University School of Medicine, Yokohama, Japan

**Keywords:** colon cancer, adjuvant chemotherapy, 12-gene recurrence score (12-RS), oncotype DX, treatment recommendation, IDEA collaboration

## Abstract

**Background:**

Recent advances in adjuvant chemotherapy for early colon cancer have widened physicians' recommendations on the regimen and duration (3 or 6 months) of the treatment. We conducted this prospective study to evaluate whether the 12-gene recurrence score (12-RS) assay affected physicians' recommendations on adjuvant treatment selection.

**Patients and methods:**

Patients with stage IIIA/IIIB or stage II colon cancer were enrolled. After the patients discussed adjuvant treatment with their treating physicians, the physicians filled in the questionnaire before assay indicating the treatment recommendation. When the 12-RS assay results were available, the physicians again filled in the questionnaire after assay. The primary endpoint was the rate of change in treatment recommendations from before to after the assay, with a threshold rate of change being 20%. Patients with stage IIIA/B to II were enrolled in a ratio of 2 : 1.

**Results:**

Overall, the treatment recommendations changed in 40% of cases after obtaining 12-RS assay results. Recommendations were changed in 45% (80/178; 95% confidence interval, 37% to 53%; *P* < 0.001) and 30% (29/97; 95% confidence interval, 21% to 40%; *P* < 0.001) of patients with stage IIIA/B and II colon cancer, respectively. Patients with stage IIIA/B cancer had significantly more change than those with stage II cancer (*P* = 0.0148). From before to after the 12-RS assay, the percentage of patients whose physicians reported being confident in their treatment recommendations significantly increased from 54% to 81% in stage IIIA/B (*P* < 0.001) and from 65% to 83% in stage II (*P* < 0.001).

**Conclusion:**

Our study confirmed the usefulness of the 12-RS assay in aiding the physician–patient decision-making process for tailoring adjuvant chemotherapy for stage IIIA/B colon cancer.

## Highlights

• This study investigated the impact of the 12-gene recurrence score (12-RS) assay on adjuvant chemotherapy decision making.
• Physicians changed treatment recommendations in 45% in stage IIIA/B and 30% in stage II patients after 12-RS assay results.
• Physician confidence increased from 54% to 81% in stage IIIA/B and from 65% to 83% in stage II after 12-RS assay results.

## Introduction

Adjuvant chemotherapy is the standard of care after complete surgical resection for stage III and high-risk stage II colon cancer.^1^, ^2^, ^3^, ^4^ Several guidelines have stated that preferred regimens involve a fluoropyrimidine-based adjuvant treatment with or without oxaliplatin.^5^^,^^6^

Conventionally, the recommended duration of adjuvant chemotherapy had been 6 months.^1^^,^^7^, ^8^, ^9^ The recent combined analysis by the International Duration Evaluation of Adjuvant Therapy (IDEA) collaboration for stage III colon cancer, including the Japanese ACHIEVE trial, suggested that shortening the oxaliplatin-based adjuvant chemotherapy may be possible. Especially, in the IDEA low-risk stage III (T1-3 and N1), 3 months of capecitabine and oxaliplatin (CAPOX) was shown to be non-inferior to 6 months. Conversely, in the IDEA high-risk stage III (T4 and/or N2), 6 months of oxaliplatin-based chemotherapy may be required.^10^, ^11^, ^12^, ^13^ This risk-stratified approach has not only widened clinicians' choice in determining an appropriate regimen and duration for each patient, but has introduced diversity into the choice of treatment.

Adjuvant treatment recommendations for patients with stage II colon cancer have been debatable.^2^^,^^8^^,^^14^, ^15^, ^16^ Several points, including whether to conduct chemotherapy, whether to use oxaliplatin in chemotherapy, and the regimen and duration of oxaliplatin-based chemotherapy, need to be taken into account. Nowadays, after the IDEA collaboration for high-risk stage II colon cancer, the choice of appropriate treatment may become even more complicated.^17^^,^^18^ A more detailed stratification of stage II and III colon cancer patients according to the risk of recurrence will allow physicians and patients to make more informed decisions towards tailoring the adjuvant regimen to refine the expectations of balancing the benefit and toxicity of chemotherapy.

The 12-gene recurrence score (12-RS; Genomic Health Inc., Redwood City, CA) assay, also known as Oncotype DX® Colon Recurrence Score, evaluates the recurrence risk of colon cancer independent of conventional prognostic parameters. The development of the 12-RS has been described and published previously.^19^ The algorithm for this 12-RS assay is based on seven cancer-related and five reference genes. A total of 761 candidate genes were selected from the published literature, genomic databases, pathway analysis, and from microarray-based gene expression profiling experiments. A real-time reverse transcriptase polymerase chain reaction method was used to quantify the expression of genes in RNA isolated from formalin-fixed, paraffin-embedded (FFPE) tumor tissue. Expression levels of these candidate genes were measured on samples from four separate studies involving patients with resected stage II and III colon cancer. The National Surgical Adjuvant Breast and Bowel Project (NSABP) C-01 and C-02 (*n* = 270) and a Cleveland Clinic observational series (*n* = 765), and two studies involving surgery followed by adjuvant fluorouracil (FU)/folinic acid (FA) chemotherapy [NSABPC-04 (*n* = 308) and NSABP C-06 (*n* = 508)] then allowed identification of a limited set of genes significantly associated with recurrence. This 12-RS assay has then been validated in four independent studies and consistently showed that the RS results correlated with the risk of recurrence for stage II and III colon cancer patients at 3 years and 5 years, regardless of the treatment.^20^, ^21^, ^22^, ^23^ The impact of the 12-RS assay on deciding adjuvant treatment between physicians and patients has been reported in four studies in stage II cancer.^24^, ^25^, ^26^, ^27^ However, the impact of the 12-RS assay on clinical decisions in stage II/III patients after the IDEA collaboration has not been elucidated.

In this paper, we report the results of a multicenter, prospective, observational study (SUNRISE-DI) to evaluate whether the 12-RS assay allowed a more detailed risk stratification of patients with stage IIIA/B and II colon cancer, helped physicians to make a recommendation for adjuvant chemotherapy, and led to tailoring the treatment of each patient in the current ‘post-IDEA collaboration’ era.

## Materials and methods

### Eligibility criteria

Inclusion criteria for participation in this study were: age ≥20 years, Eastern Cooperative Oncology Group (ECOG) performance score 0-1, colon cancer stage II or IIIA/IIIB [Union Internationale Contre le Cancer tumor–node–metastasis (UICC TNM) stage 8th edition],^28^ completed R0 tumor resection, candidate for fluoropyrimidine-based ± oxaliplatin treatment starting ≤8 weeks after surgery, willingness to consider adjuvant chemotherapy based on the 12-RS results, and sufficient primary tumor tissue available for testing by Oncotype DX® Pathology Guidelines. Patients were excluded if they had any of the following: deficient mismatch repair (MMR) (high microsatellite instability) at enrollment, known dihydropyrimidine dehydrogenase deficiency, prior exposure to a fluoropyrimidine or oxaliplatin, previous radiation therapy of tumor site, or double cancer. A signed informed consent form was obtained from all patients before enrollment to this study. The scientific and ethical aspects of the study were reviewed and approved by the institutional review board in each participating institution. The study was conducted according to the principles of the Declaration of Helsinki and was registered in the UMIN Clinical Trials Registry (UMIN000028784).

### 12-RS assay

FFPE primary cancer tissue from patients selected for inclusion in the study was sent to a central laboratory of Genomic Health Inc. and the 12-RS assay was carried out without knowledge of clinical factors or outcomes. Prespecified genes and a validated algorithm were used to calculate RS in each patient. Risk groups were defined based on the 12-RS results as low (RS ≤ 29), intermediate (30 ≤ RS ≤ 40), and high (RS ≥ 41).

### Study procedures

Eligible patients with stage II or IIIA/IIIB colon cancer who had complete surgical tumor resection were invited to discuss the recommendation on adjuvant therapy with their treating physicians before carrying out the 12-RS assay and asked for their consent to participate in the study. The treatment recommendation was documented, and the physician ordered the 12-RS assay and filled in the questionnaire before assay indicating the treatment recommendation and the physician's confidence in it on a five-tier scale of ‘not confident at all,’ ‘somewhat not confident,’ ‘neutral,’ ‘confident,’ and ‘very confident.’

After availability of the 12-RS assay results (usually 17-21 days later), the treatment recommendation was again discussed between the physician and the patient. Once more, the recommendation was documented, and the physician then filled in the questionnaire after assay indicating the treatment recommendation after receiving the 12-RS assay results and the level of confidence towards the new recommendation. How the treatment strategy was decided according to the 12-RS assay results is shown in Supplementary Table S1, available at https://doi.org/10.1016/j.esmoop.2021.100146.

### Study endpoints

The primary endpoint was the rate of change in treatment recommendations from before to after the assay. Relevant changes were defined as (i) add/omit oxaliplatin to/from a fluoropyrimidine-based chemotherapy, (ii) change oxaliplatin treatment duration (3 to 6 months, or vice versa), and (iii) change from use of any chemotherapy to no chemotherapy or vice versa. The secondary endpoints included analyses of the aforementioned changes according to tumor stage (stage II and IIIA/B) and 12-RS-defined risk groups. For stage IIIA/B, analyses according to risk groups as defined by the IDEA collaboration (low risk, T1-3N0; high-risk, T4 or N2) were also conducted. A change in the confidence level of physicians before and after the assay was evaluated.

### Statistics

The primary objective was to estimate the rate of change in treatment recommendations from before to after the assay. With an expected change rate in treatment recommendations of 25%, a total of 300 patients were planned for achieving a target half width of <5% for the two-sided 95% confidence interval (CI). Patients with stage II to IIIA/B were enrolled in a ratio of 1 : 2. The rates of change from before to after the assay were compared with a threshold change rate of 20% by a one-arm binomial test. The McNemar test was used to evaluate changes in physician confidence levels. The rates between two independent groups were compared by the chi-square test. All *P* values were reported as two-tailed, and *P* < 0.05 was considered statistically significant.

## Results

### Patient disposition

A total of 305 patients from 14 centers in Japan were registered between November 2017 and January 2019. Of these, 11 patients were excluded before the pre-assay questionnaire due to ineligible staging (*n* = 9), main tumor location in the rectum (*n* = 1), and double cancer (*n* = 1); 19 patients were excluded due to start of adjuvant chemotherapy before availability of the 12-RS results (*n* = 8); tumor sampling failure (*n* = 3); inappropriateness to continue the study (*n* = 1), patient withdrawal (*n* = 1); and deficient MMR known before post-assay assessment (*n* = 6). Thus 275 patients were eligible for analyses (Supplementary Figure S1, available at https://doi.org/10.1016/j.esmoop.2021.100146).

### Patient characteristics

Of the 275 patients, 97 (35.3%) had stage II and 178 (64.7%) had stage IIIA/B tumors. The median age was 70 and 68 years in patients with stage II and stage IIIA/B tumors, respectively. T4 and inadequate nodal harvest were seen in 23.7% and 5.2% of patients with stage II and 23.0% and 7.9% of those with stage IIIA/B cancer. Among patients with stage IIIA/B cancer, 67.4% were deemed to be at low risk and 32.6% at high risk of recurrence according to the IDEA classification. The 12-RS results classified 82.5% of 97 stage II and 81.5% of 178 stage IIIA/B patients as low RS (<30) (Table 1).

**Table 1:** Baseline patients characteristics

	Stage II (*N* = 97)	Stage IIIA/B (*N* = 178)	Overall (*N* = 275)
Age (years), median (range)	70 (37-90)	68 (25-89)	69 (25-90)
Sex, male, *n* (%)	48 (49.5)	69 (38.8)	117 (42.5)
Primary site of tumor, *n* (%)			
Right colon (cecum to transverse)	46 (47.4)	69 (38.8)	115 (41.8)
Left colon (descending to rectosigmoid)	51 (52.6)	109 (61.2)	160 (58.2)
pT stage, *n* (%)			
T1	0	4 (2.2)	4 (1.5)
T2	0	20 (11.2)	20 (7.3)
T3	74 (76.3)	113 (63.5)	187 (68.0)
T4	23 (23.7)	41 (23.0)	64 (23.3)
pN stage, *n* (%)			
N0	97 (100)	0	97 (35.3)
N1	0	161 (90.4)	161 (58.5)
N2	0	17 (9.6)	17 (6.2)
Poorly differentiated, *n* (%)	2 (2.1)	11 (6.2)	13 (4.7)
Inadequate nodal harvest, *n* (%)	5 (5.2)	14 (7.9)	19 (6.9)
Lymphatic invasion, *n* (%)	45 (46.4)	112 (62.9)	157 (57.1)
Vascular invasion, *n* (%)	59 (60.8)	124 (69.7)	183 (66.5)
Perineural invasion, *n* (%)	31 (32.0)	63 (35.4)	94 (34.2)
Recurrence score (RS), *n* (%)			
<30 (low)	80 (82.5)	145 (81.5)	225 (81.8)
30-40 (intermediate)	15 (15.5)	25 (14.0)	40 (14.5)
≥40 (high)	2 (2.1)	8 (4.5)	10 (3.6)
Risk groups as defined in IDEA, *n* (%)			
Low-risk (T1-3 and N1)	N/A	120 (67.4)	N/A
High-risk (T4 and/or N2)	N/A	58 (32.6)	N/A

IDEA, International Duration Evaluation of Adjuvant Therapy; N/A, not applicable.

### Primary endpoint: rate of change in treatment recommendations

**Figure 1:**
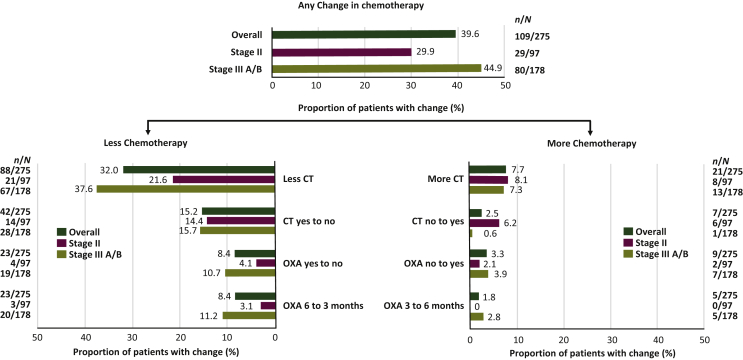
Changes in treatment recommendations after versus before availability of the 12-gene recurrence score (12-RS) results in the overall population and according to tumor stage. CT, chemotherapy; OXA, oxaliplatin.

After availability of the 12-RS results, the treating physicians significantly changed their treatment recommendations in 39.6% of patients (109/275; 95% CI, 34% to 45%; one-arm binomial *P* < 0.001) (Figure 1). Some 32.0% (88/275; 95% CI, 26.5% to 37.9%) of patients then received a recommendation to have less treatment, i.e. their recommendation was changed from any chemotherapy to no chemotherapy (15.2%), from use of oxaliplatin to no oxaliplatin (8.4%), or from 6 months to 3 months of oxaliplatin-based therapy (8.4%). Conversely, 7.6% (21/275; 95% CI, 4.8% to 11.4%) were recommended to have more intensive treatment (no oxaliplatin to use of oxaliplatin, 3 months to 6 months of oxaliplatin-based therapy, or no chemotherapy to any chemotherapy).

### Rates of change in treatment recommendations by tumor stage

The treating physicians significantly changed their treatment recommendations in patients with stage IIIA/B cancer after availability of the 12-RS results [44.9% (80/178); 95% CI, 37.5% to 52.6%; *P* < 0.001] (Figure 1). Similarly, the treating physicians significantly changed their treatment recommendations in patients with stage II cancer [29.9% (29/97); 95% CI 21.0% to 40.0%; *P* < 0.001]. Patients with stage IIIA/B cancer had significantly more change than those with stage II cancer (*P* = 0.0148). In both stage IIIA/B (37.6% versus 7.3%) and stage II (21.6% versus 8.2%), more patients received a change in recommendation towards less intensive therapy than towards more intensive therapy. Table 2 summarizes the proportion of stage II patients who had a change in treatment recommendations according to 12-RS-defined risk groups.

**Table 2:** Changes in treatment recommendations after versus before availability of the 12-gene recurrence score (12-RS) results according to RS category in patients with stage II disease

	Any RS (*n* = 97)	RS < 30 (*n* = 80)	RS ≥ 30 (*n* = 17)
Recommendation to less intensive treatment, *n* (%)	21 (21.6)	21 (26.3)	0 (0)
Oxaliplatin yes to no	4 (4.1)	4 (5.0)	
Oxaliplatin 6 months to 3 months	3 (3.1)	3 (3.8)	
Any chemotherapy to no chemotherapy	14 (14.4)	14 (17.5)	
Recommendation to more intensive treatment, *n* (%)	8 (8.2)	5 (6.3)	3 (17.6)
Oxaliplatin no to yes	2 (2.1)	1 (1.3)	1 (5.9)
Oxaliplatin 3 months to 6 months			
No chemotherapy to any chemotherapy	6 (6.2)	4 (5.0)	2 (11.8)
Any change in treatment recommendation, *n* (%)	29 (29.9)	26 (32.5)	3 (17.6)
No change in treatment recommendation, *n* (%)	68 (70.1)	54 (67.5)	14 (82.4)

These results held the same trend in the 12-RS-defined low-risk group in each stage because the majority of patients (82.5% in stage II and 81.5% in stage IIIA/B) are those who had a low RS (<30).

We observed patients who had a treatment recommendation that did not match the results of 12-RS. In total, 5.6% (10/178) and 5.2% (5/97) had unexpected changes in treatment recommendations at stage IIIA/B and stage II, respectively. Supplementary Table S2, available at https://doi.org/10.1016/j.esmoop.2021.100146 summarizes the list of those patients who had an unexpected change of treatment recommendation.

### Rates of change in treatment recommendations in stage IIIA/B patients by IDEA risk groups

Table 3 summarizes the proportion of stage IIIA/B patients who had a change in treatment recommendations according to IDEA risk groups and 12-RS-defined risk groups. The 12-RS results led to changes of treatment recommendations in 48.3% (58/120; 95% CI, 39.1% to 57.6%; *P* < 0.001) of patients with IDEA low-risk cancer and in 37.9% (22/58; 25.5% to 51.6%; *P* < 0.001) of those with IDEA high-risk cancer. The most common change in the IDEA low-risk group was a change from chemotherapy to no chemotherapy (19.2%), followed by from oxaliplatin to no oxaliplatin (12.5%), and shortening oxaliplatin therapy from 6 to 3 months (9.2%). In the IDEA high-risk group, the most common change was shortening oxaliplatin treatment (15.5%), followed by chemotherapy to no chemotherapy (8.6%), and oxaliplatin to no oxaliplatin (6.9%).

**Table 3:** Changes in treatment recommendations after versus before availability of the 12-gene recurrence score (12-RS) results according to IDEA risk group and RS category in patients with stage IIIA/B disease

	IDEA low risk			IDEA high risk		
	Any RS (*n* = 120)	RS < 30 (*n* = 105)	RS ≥ 30 (*n* = 15)	Any RS (*n* = 58)	RS < 30 (*n* = 40)	RS ≥ 30 (*n* = 18)
Recommendation to less intensive treatment, *n* (%)	49 (40.8)	46 (43.8)	3 (20.0)	18 (31.0)	17 (42.5)	1 (5.6)
Oxaliplatin yes to no	15 (12.5)	14 (13.3)	1 (6.7)	4 (6.9)	4 (10.0)	
Oxaliplatin 6 months to 3 months	11 (9.2)	10 (9.5)	1 (6.7)	9 (15.5)	9 (22.5)	
Any chemotherapy to no chemotherapy	23 (19.2)	22 (21.0)	1 (6.7)	5 (8.6)	4 (10.0)	1 (5.6)
Recommendation to more intensive treatment, *n* (%)	9 (7.5)	4 (3.8)	5 (33.3)	4 (6.9)	2 (5.0)	2 (11.1)
Oxaliplatin no to yes	4 (3.3)	4 (3.8)		3 (5.2)	1 (2.5)	2 (11.1)
Oxaliplatin 3 months to 6 months	5 (4.2)		5 (33.3)			
No chemotherapy to any chemotherapy, *n* (%)				1 (1.7)	1 (2.5)	
Any change in treatment recommendation, *n* (%)	58 (48.3)	50 (47.6)	8 (53.3)	22 (37.9)	19 (47.5)	3 (16.7)
No change in treatment recommendation, *n* (%)	62 (51.7)	55 (52.4)	7 (46.7)	36 (62.1)	21 (52.5)	15 (83.3)

IDEA, International Duration Evaluation of Adjuvant Therapy.

Of the IDEA low-risk patients, 105 had an RS < 30 and physicians changed their recommendation to less intensive treatment in 43.8% (46/105; 95% CI, 34.1% to 53.8%) of patients, whereas of 15 patients who had an RS ≥ 30, changes to less intensive therapy were less common [3.8% (3/15); 95% CI, 4.3% to 48.1%]. Of the IDEA high-risk patients, 40 had an RS < 30 and physicians changed their recommendations to less intensive treatment in 42.5% (17/40; 95% CI, 27.0% to 59.1%) of patients, whereas of 18 patients who had an RS ≥ 30, there was only one change to less intensive therapy [5.6% (1/18); 95% CI, 0.1% to 27.3%].

### Rates of change in treatment recommendations by age

One hundred and fifty patients (54.5%) were <70 years old and 125 (45.5%) were ≥70 years old. The treating physicians significantly changed their treatment recommendations in patients <70 years old after availability of the 12-RS results [44.0% (66/150); 95% CI, 35.9% to 52.3%; *P* < 0.001]. Similarly, the treating physicians significantly changed their treatment recommendations in patients ≥70 years old [34.4% (43/125); 95% CI, 26.1% to 43.4%; *P* < 0.001]. In both age groups, <70 (35.3% versus 8.7%) and ≥70 (28.0% versus 6.4%), more patients received a change in recommendation towards less intensive therapy than towards more intensive therapy. The change of treatment recommendation was not significantly different between the two age groups (34.4% versus 44.0%, *P* = 0.105).

### Changes in confidence levels of physicians

After 12-RS testing, the percentage of patients whose physicians reported being ‘confident’ or ‘strongly confident’ in their treatment recommendations significantly increased from 57.8% to 82.2% (McNemar, *P* < 0.001) (Table 4). In the stage II subpopulation, this percentage increased from 64.9% to 83.5% (*P* < 0.001), and in the stage IIIA/B population from 53.9% to 81.5% (*P* < 0.001).

**Table 4:** Changes in physicians' level of confidence in the treatment recommendation

Overall *n* = 275	After 12-RS testing not confident^a^	After 12-RS testing confident^b^	Total
Before 12-RS testing not confident^a^, *n* (%)	26 (9.5)	90 (32.7)	
Before 12-RS testing confident^b^, *n* (%)	23 (8.4)	136 (49.5)	159 (57.8)
Total, *n* (%)		226 (82.2)	

Stage II *n* = 97	After 12-RS testing not confident^a^	After 12-RS testing confident^b^	Total
Before 12-RS testing not confident^a^, *n* (%)	6 (6.2)	28 (28.9)	
Before 12-RS testing confident^b^, *n* (%)	10 (10.3)	53 (54.6)	63 (64.9)
Total, *n* (%)		81 (83.5)	

Stage IIIA/B *n* = 178	After 12-RS testing not confident^a^	After 12-RS testing confident^b^	Total
Before 12-RS testing not confident^a^, *n* (%)	20 (11.2)	62 (34.8)	
Before 12-RS testing confident^b^, *n* (%)	13 (7.3)	83 (46.6)	96 (53.9)
Total, *n* (%)		145 (81.5)	

12-RS, 12-gene recurrence score.

a Not confident: ‘not confident at all’, ‘somewhat not confident’, and ‘neutral’.

b Confident: ‘confident’ and ‘very confident’.

## Discussion

This is the first study that prospectively evaluated the impact of the 12-RS assay on the physician-patient decision-making process for stage III colon cancer patients in the era after the IDEA collaboration. Our results demonstrated that the 12-RS assay significantly affected physicians' treatment recommendations in patients with stage IIIA/B as well as with stage II colon cancer; a comparison before and after the assay showed that treatment recommendations were changed in 44.9% of stage IIIA/B (80/178; *P* < 0.001) and 29.9% of stage II patients (29/97; *P* < 0.001), respectively, after availability of the 12-RS result. It is of note that the difference of change rates was statistically significant between the two stages (44.9% versus 29.9%; *P* < 0.001).

Of all the changes in stage IIIA/B, 37.6% (67/178) were towards less treatment and, of particular interest, 11.2% (20/178) of change was a switch from 6-months to 3-months CAPOX. In stage II, 21.6% (21/97) was toward less treatment and a switch from 6-months to 3-months CAPOX was seen in only 3.1% (3/97) of the patients.

The analyses according to risk groups as defined by the IDEA collaboration for stage III found that a low RS result (RS < 30) affected treatment recommendations for both IDEA high-risk and low-risk stage IIIA/B patients similarly; in the IDEA low-risk stage IIIA/B patients, physicians recommended using less treatment after obtaining a low RS result in nearly half (46/105; 43.8%) of patients. In the smaller group of IDEA high-risk stage IIIA/B patients with a low RS result, physicians also tended to recommend less treatment (17/40; 42.5%). These results suggest that a more detailed stratification of stage IIIA/B patients for tailoring the adjuvant regimen may be possible by the 12-RS assay.

The effect of adjuvant chemotherapy with oxaliplatin for older patients (≥70 years old) is unclear, and the age of the patients is one of the important factors. In this study, the change of treatment recommendation was not significantly different between patients aged <70 years and patients aged ≥70 years (34.4% versus 44.0%, *P* = 0.105). 12-RS results may affect treatment recommendations regardless of age.

The proportion of physicians being ‘confident’ or ‘very confident’ of their treatment recommendations increased from 64.9% to 83.5% in stage II after availability of the 12-RS assay (*P* < 0.001). The proportion was more pronounced in physicians treating stage IIIA/B patients with nearly a 30% increase (53.9% versus 81.4%; *P* < 0.001), although treatment options have been increased since the results of IDEA collaboration became available.^10^, ^11^, ^12^, ^13^

These results strongly suggest the usefulness of 12-RS in aiding the physician–patient decision-making process in stage IIIA/B colon cancer, although the 12-RS has been originally validated in stage II patients and there have been only two validation studies for stage III, including our SUNRISE study.^22^^,^^23^

There are several limitations in this study. First, the number of patients with an RS ≥ 30 was small in the current SUNRISE-DI study. The distribution of RS in this study was different from that observed in the previous SUNRISE study^23^; the proportions of the low, intermediate, and high-risk groups defined by 12-RS results were, respectively, 82%, 15%, and 2% in this study and 60%, 26%, and 14% in the SUNRISE study for stage II, and 81%, 14%, and 4% in this study and 46%, 31%, and 23% in the SUNRISE study for stage IIIA/B. However, the patient distribution in the SUNRISE study may not accurately reflect the actual distribution, as it is intended for patients who have not received adjuvant chemotherapy and is also for randomly selected patients. In this SUNRISE-DI study, since patients are enrolled prospectively, it is considered that the patient distribution is closer to the actual one. The sample size of patients with an RS < 30 enabled the SUNRISE-DI study to show the clinical utility of 12-RS in patients with a low RS score, whereas the limited number of patients with an RS ≥ 30 restricts the reliability of the results in this population. Second, we observed patients who had a treatment recommendation that did not match the results of 12-RS. Supplementary Table S1, available at https://doi.org/10.1016/j.esmoop.2021.100146 summarizes the list of those patients who had an unexpected change of treatment recommendation. For example, the first patient in the list was IDEA low-risk stage III with a T3N1M0 tumor and the treatment recommendation was 6 months of 5-FU plus leucovorin. After the results of 12-RS became available, the treatment recommendation was changed to 6 months of CAPOX, although her RS score was 18 (<30), a relatively low risk of recurrence, and she declared she was a ‘fatalist’ (see the footnote in the Supplementary Table S1, available at https://doi.org/10.1016/j.esmoop.2021.100146 for the definition of ‘fatalist’). The turnaround time for the 12-RS is generally 17-21 days and patients may have time to consider an appropriate treatment during that period, resulting in unexpected changes. In total, 5.6% (10/178) and 5.2% (5/97) had an unexpected change of treatment recommendation in stage IIIA/B and stage II, respectively. Third, the patient registration in this study had started before the results of the IDEA collaboration for high-risk stage II became available, although it started after availability of the result for stage III. Hence, our results for stage II may not reflect the effect of the IDEA collaboration for high-risk stage II appropriately. Fourth, our results did not include efficacy results such as disease-free survival (DFS). The real impact of the 12-RS assay cannot be accurately assessed without showing how it affects long-term prognosis as a result of changing the recommended treatment by the 12-RS results. Therefore, it is planned to conduct a follow-up on the 3-year DFS. This result will be published in the future.

Despite those limitations, the SUNRISE-DI study confirmed the usefulness of the 12-RS assay in aiding the physician–patient decision-making process and physicians' acceptance of the 12-RS in patients with stage IIIA/B colon cancer after availability of the results of IDEA collaboration. The 12-RS also provided valuable support to physicians in treating patients with stage II. However, further long-term follow-up is needed in order to confirm the clinical utility of the 12-RS assay.

### Conclusion

In conclusion, our study showed that the 12-RS could contribute to making more informed decisions towards tailoring the adjuvant regimen to refine the expectations of balancing the benefit and toxicity of chemotherapy. New risk assessment technologies such as circulating tumor DNA analysis evaluating minimal residual disease are evolving, but have not been firmly established yet. Combining these new modalities and the 12-RS may offer enhanced risk assessment accuracy in the future.
