# Supplementary material for: Impact of the 12-gene recurrence score assay on deciding adjuvant chemotherapy for stage II and IIIA/B colon cancer: the SUNRISE-DI study
Source: ESMO Open. 2021 May 10;6(3):100146. doi: 10.1016/j.esmoop.2021.100146 (PMC8134704; doi:10.1016/j.esmoop.2021.100146)
Supplement: Supplementary Material [file mmc1.docx]

**Supplementary figure 1 (online only). Consort diagram.**

**Registered patients**

**(n=305)**

From Nov. 2017 to Jan. 2019, 14 Centers

**Patients with pre-assay questionnaire (n=294)**

**Patients with post–assay questionnaire (n=275)**

**Full Analysis Set**

**Excluded before pre-assay** **questionnaire (n=11)**

- Staging ineligible (n=9)
- Main tumor located at rectum (n=1)
- Double cancer (n=1)

**Excluded before post-assay** **questionnaire (n=19);**

**patients meet discontinuation criteria**

- Patients needed adjuvant chemotherapy before obtaining the RS results (n=8)
- Sample failures (n=3)
- Inappropriate to continue the study (n=1)
- Patient‘s withdrawal (n=1)
- Known dMMR before post-assay questionnaire (n=6)

**Supplementary Table 1 (online only). How did the physicians decide the treatment strategy according to recurrence score (RS).**

| **Stage** | **treatment strategy** |
| --- | --- |
| **Stage IIIA/B** |  |
| RS < 30 | Less intensive treatment should be considered. |
| RS ≥ 30 | More intensive treatment should be considered. |
| **Stage II** |  |
| RS < 30 | Less intensive treatment should be considered. |
| RS ≥ 30 | More intensive treatment should be considered. |

RS, Recurrence Score

**Supplementary Table 2 (online only). The list of 15 patients who had an unexpected change of treatment recommendation after availability of 12-RS result.**


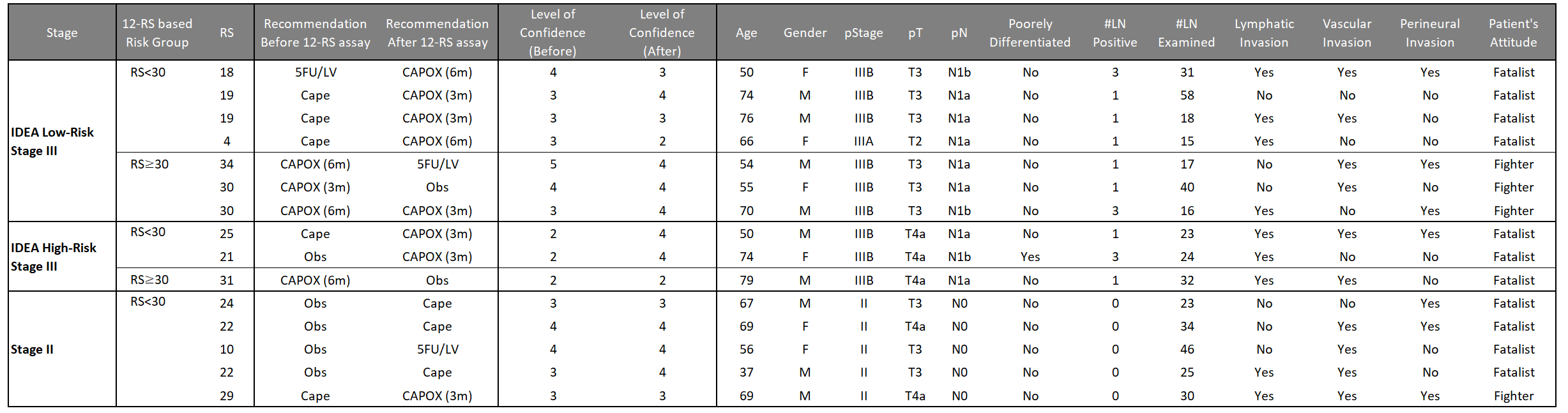


RS, Recurrence Score; Cape, Capecitabine; 3m, 3 months; 6m, 6 months; Obs, Observation; LN, Lymph Nodes; #, Number of;

“Fighter” was defined as a patient willing to sacrifice no more than 2% benefit to reduce toxicity; others were defined as “fatalist”.
